# Mediating Role of the Reward Network in the Relationship between the Dopamine Multilocus Genetic Profile and Depression

**DOI:** 10.3389/fnmol.2017.00292

**Published:** 2017-09-14

**Authors:** Liang Gong, Cancan He, Yingying Yin, Hui Wang, Qing Ye, Feng Bai, Yonggui Yuan, Haisan Zhang, Luxian Lv, Hongxing Zhang, Zhijun Zhang, Chunming Xie

**Affiliations:** ^1^Department of Neurology, Affiliated ZhongDa Hospital, School of Medicine, Southeast University Nanjing, China; ^2^Department of Psychology, Affiliated ZhongDa Hospital, School of Medicine, Southeast University Nanjing, China; ^3^Neuropsychaitric institute, Affiliated ZhongDa Hospital, Southeast University Nanjing, China; ^4^Department of Psychiatry, Henan Mental Hospital, the Second Hospital of Xinxiang Medical University Xinxiang, China

**Keywords:** depression, dopamine, imaging genetics, reward network, multilocus genetic profile

## Abstract

Multiple genetic loci in the dopamine (DA) pathway have been associated with depression symptoms in patients with major depressive disorder (MDD). However, the neural mechanisms underlying the polygenic effects of the DA pathway on depression remain unclear. We used an imaging genetic approach to investigate the polygenic effects of the DA pathway on the reward network in MDD. Fifty-three patients and 37 cognitively normal (CN) subjects were recruited and underwent resting-state functional magnetic resonance imaging (R-fMRI) scans. Multivariate linear regression analysis was employed to measure the effects of disease and multilocus genetic profile scores (MGPS) on the reward network, which was constructed using the nucleus accumbens (NAc) functional connectivity (NAFC) network. DA-MGPS was widely associated within the NAFC network, mainly in the inferior frontal cortex, insula, hypothalamus, superior temporal gyrus, and occipital cortex. The pattern of DA-MGPS effects on the fronto-striatal pathway differed in MDD patients compared with CN subjects. More importantly, NAc-putamen connectivity mediates the association between DA MGPS and anxious depression traits in MDD patients. Our findings suggest that the DA multilocus genetic profile makes a considerable contribution to the reward network and anxious depression in MDD patients. These results expand our understanding of the pathophysiology of polygenic effects underlying brain network abnormalities in MDD.

## Introduction

Major depressive disorder (MDD) is a serious disease that poses major risks to human physical and mental health and will become the world's most common and economically burdensome illness by 2030 (Mathers and Loncar, 2006). Although there are some effective antidepressant treatments (Girardi et al., 2009; Murrough et al., 2013), the etiology and pathophysiology of MDD remain obscure. Depression is heritable (~35%) (Sullivan et al., 2000); however, the neural mechanisms of how risk genetic loci contribute to depression are still unclear. Accumulating evidence implicates the dopaminergic system in the pathophysiology of depression and antidepressant efficacy (Papakostas, 2006; Nelson et al., 2012; Dell'Osso et al., 2013). This suggests that depression is at least partly due to a decrease in extracellular dopamine (DA) levels (Lambert et al., 2000; Leggio et al., 2013). Thus, DA-related genetic variants that modulate endogenous dopaminergic neurotransmission might significantly contribute to depression pathophysiology. Although the results are not completely consistent, several studies have found that several DA-related genetic variants including catechol-O-methyltransferase (*COMT*), monoamine oxidase A (*MAOA*), DA D2 receptor (*DRD2*), and DA transporter gene (*DAT*) are closely associated with depression and antidepressant responses (Baune et al., 2008; Dong et al., 2009; Opmeer et al., 2010; Xu et al., 2011; Dannlowski et al., 2013). Specifically, Val alleles of the *COMT* gene polymorphism (rs4680) substantially increase COMT activity, improve DA metabolism and transmission, decrease prefrontal extracellular DA, and are associated with better processing of aversive stimuli (Stein et al., 2006; Winterer et al., 2006; Wu et al., 2012; Antypa et al., 2013). The G allele of the *MAOA* gene polymorphism (rs6323) encodes the higher activity form of the enzyme and thereby decreases dopaminergic tone, and is significantly associated with a poor antidepressant response in MDD patients (Shih et al., 1999; Ducci et al., 2006; Leuchter et al., 2009; Xu et al., 2011). Interestingly, the *DRD2* and *DRD3* genes are predominantly involved in the ventral striatum. For example, the C allele of the *DRD2* gene polymorphism (rs6277) is associated with low striatal DRD2 availability and affects receptor affinity, with higher levels of depressive rumination in depressed individuals (Hirvonen et al., 2004, 2009; Whitmer and Gotlib, 2012). DRD3 is a DA receptor with distinct high distribution in the limbic system, especially the nucleus accumbens (NAc). Importantly, the Gly variant of the *DRD3* gene polymorphism (rs6280) has 4–5 times greater affinity for DA compared to the Ser variant, and subjects with this form exhibit increased striatal reward-related DA release during a gambling task (Jeanneteau et al., 2006; Savitz et al., 2013). Collectively, these findings suggest that the multiple genetic effects of the DA pathway play a critical role in the pathophysiology of depression.

Depression is likely influenced by the joint influences of multiple genes operating within specific biological pathways (Woody and Gibb, 2015). Recently, the multilocus genetic profile score (MGPS) approach was employed to explore polygenic effects on psychiatric disorders (Bogdan et al., 2017). The MGPS approach summates several gene polymorphisms variants in a given functional neural system to obtain a composite of relative signaling (Nikolova et al., 2011; Bogdan et al., 2016). Researchers have begun to investigate the cumulative genetic effects of the DA pathway on depression (Stice et al., 2012; Pearson-Fuhrhop et al., 2014) by constructing MGPS from several gene variants involved in DA metabolism and signaling. These studies have revealed that lower MGPS reflecting DA signaling capacity predicts more severe depressive symptoms in healthy adults and depression patients (Pearson-Fuhrhop et al., 2014). On the other hand, neuroimaging genetics studies have demonstrated that the multilocus genetic composite of the DA pathway, but not a single single-nucleotide polymorphism (SNP) genotype, can predict reward circuitry activity during reward paradigm tasks (Nikolova et al., 2011; Stice et al., 2012). However, the cumulative effect of dopaminergic pathway genes on the intrinsic reward network in MDD patients is still unclear.

In the present study, we aimed to investigate the neural mechanism underlying the polygenic effects of the DA pathway on the reward network [constructed using the NAc functional connectivity (NAFC) network] in MDD patients. We hypothesized that the DA-MGPS would be significantly associated with the reward network and depressive symptoms and that dysfunctional connectivity within the reward network would mediate the association between DA-MGPS and depression in MDD patients.

## Materials and methods

### Participants

All participants came from a large sample of the Project of Risk Gene and Pharmacogenomics for Depression and underwent exome sequencing based on biological pathways. A total of 100 subjects (40 CN and 60 MDD) were enrolled and completed resting-state functional MRI (R-fMRI) scans as part of the current study. All CN subjects were recruited through community health screening and media advertisements. The MDD patients were recruited from the outpatient and inpatient populations at the Department of Psychiatry, Affiliated ZhongDa Hospital of Southeast University. All subjects were Chinese Han and right handed. This study was approved by the Research Ethical Committee of Affiliated ZhongDa Hospital of Southeast University, and an informed consent form was signed by all participants. Because of excessive head motion and/or incomplete EPI image scans, seven MDD and three CN subjects were excluded. The remaining 53 MDD and 37 CN subjects were entered into the final imaging genetic analysis.

### Inclusion and exclusion criteria

The inclusion criteria for MDD: (1) met the diagnostic criteria for MDD according to the Diagnostic Statistical Manual of Mental Disorder, Fourth Edition (DSM-IV); (2) the Hamilton Rating Scale for Depression (HAMD) score was ≥17; (3) naïve to antidepressant medication or had undergone a washout period of at least five half-lives of previously prescribed medicine; 4) age 18–59 and age of onset <55 years. Exclusion criteria for MDD were: (1) other major psychiatric disorders or neurodegenerative illness history, except anxiety in the current state; (2) substance abuse history (caffeine, nicotine, and alcohol); or (3) contraindication to MRI scanning. CN subjects were required to have a Mini-mental State Examination (MMSE) score ≥26 and HAMD-17 score ≤ 7. Exclusion criteria for CN subjects were: history of neuropsychiatric disease, head injury, and drug or alcohol abuse.

### Behavior assessment

All subjects underwent a battery of neuropsychological tests including the HAMD for depression severity and the Hamilton Rating Scale for Anxiety (HAMA) for anxiety evaluation (Hamilton, 1959, 1960). We also counted the five subscores of the HAMD including anxiety/somatization, weight, cognitive disturbance, retardation, and sleep disruption (Cleary and Guy, 1977).

### Gene selection and DA variant classification

The MGPS employed in the current study captures genetic variation in several aspects of the brain's DA system, including synaptic DA availability (*COMT* and *MAOA*) and DA receptor binding (*DRD2* and *DRD3*). As descripted in the introduction, all four loci in the genetic profile were carefully selected based on their previously described links with functional changes in dopaminergic transmission and/or ventral striatum reactivity. (1) *COMT* (rs4680): the COMT enzyme, especially the Val allele, plays a particularly crucial role in regulating prefrontal DA (Winterer et al., 2006). Therefore, 1 point was added to each individual's MGPS for the presence of Met/Met, 0.5 points for Val/Met, and 0 points for Val/Val. (2) *DRD2* (rs6277): the C957T polymorphism can alter the translation and stability of DRD2, and the C allele of the C957T SNP is associated with low striatal DRD2 availability (Hirvonen et al., 2004, 2009). Thus, 0.5 points was added to the MGPS for C/T presence and 0 points for C/C presence. There was no individual with a T/T genotype in the present study. (3) *DRD3* (rs6280): DRD3 is a DA receptor that has an SNP resulting in a Ser to Gly substitution. So, 1 point was added to each individual's MGPS for Gly/Gly, 0.5 points for Ser/Gly, and 0 points for Ser/Ser. (4): *MAOA* (rs6323): The G allele of *MAOA* gene encodes the MAOA enzyme, which decreases dopaminergic tone (Leuchter et al., 2009), and has been associated with poor antidepressant response in MDD patients (Xu et al., 2011). Accordingly, 1 point was added to each individual's MGPS for T/T, 0.5 points for G/T, and 0 points for G/G.

### Genotyping

DNA was extracted from the blood using standard protocols. DNA genotyping was performed by Genesky Biotechnologies (Shanghai, China). Genotypes of selected single-nucleotide polymorphisms in *MAOA* (rs6323), *COMT* (rs4680), *DRD2* (rs6277), and *DRD3* (rs6280) genes were determined using predesigned Illumina next sequencing and array technologies (Illumina Inc., San Diego, CA, USA). Hardy–Weinberg equilibrium (HWE) tests, linkage disequilibrium statistics, and allele and genotype frequencies were calculated using PLINK 1.9 software (Purcell et al., 2007). The four SNPs did not deviate from HWE (*P* > 0.1).

### Multilocus genetic profile scores

We calculated the MGPS reflecting four variants previously associated with dopaminergic neurotransmission. Participants were scored according to the DA neurotransmission genotypes; across all loci, genotypes associated with relative increases, decreases, and intermediated effects in DA neurotransmission were assigned a score of 1, 0, and 0.5, respectively. The scores for each locus were added to create an individual profile score (Table S1). This detailed method has been described in previous studies (Stice et al., 2012; Pearson-Fuhrhop et al., 2014).

### MRI data acquisition

Imaging was performed using a Siemens Verio 3.0 Tesla scanner (Siemens, Erlangen, Germany) with a homogeneous birdcage head coil at the Affiliated ZhongDa Hospital of Southeast University, including high-resolution spoiled gradient-recalled echo (SPGR) 3D axial images and R-fMRI scans. During the data scans, all subjects were instructed to relax with their eyes closed, and stabilizers were used to immobilize the heads of the subjects. The SPGR parameters were repetition time (TR) = 1,900 ms, echo time (TE) = 2.48 ms, flip angle (FA) = 9°, acquisition matrix = 256 × 256, field of view (FOV) = 240 × 240 mm, thickness = 1.0 mm, gap = 0 mm, number of slices = 176, and number of excitations (NEX) = 1.0. Axial R-fMRI datasets were obtained in 8 min with a gradient-recalled echo-planar imaging (GRE-EPI) pulse sequence. The R-fMRI imaging parameters included TR = 2,000 ms, TE = 25 ms, FA = 90°, acquisition matrix = 64 × 64, FOV = 240 × 240 mm, thickness = 4.0 mm, gap = 0 mm, NEX = 1.0, and number of slices = 36.

### fMRI data preprocessing

The functional data were preprocessed using SPM8 toolkit (http://www.fil.ion.ucl.ac.uk/spm) and MATLAB version 7.10 (The MathWorks, Inc., Natick, MA, USA). fMRI images were preprocessed in the following manner: The first 10 volumes of the scanning session were discarded for T1 equilibration effects. The remaining 230 volumes were corrected for slice timing, realigned, and subsequently spatially normalized to the standard Montreal Neurological Institute (MNI) EPI template using the default settings. To further reduce the effects of confounding factors, six motion parameters, the global mean signal, white matter (WM) signal, and cerebrospinal fluid (CSF) signal were removed from the data through linear regression (Chen et al., 2012). Participants with head motion >2 mm in any direction of x, y, and z or 2 degrees of any angular motion were excluded. No significant difference in head motion was observed among the six groups (*P* > 0.05). Moreover, a band-pass filter was applied to maintain low-frequency fluctuations within a frequency range of 0.015–0.1 Hz.

### Structural image analysis

To avoid the bias of functional connectivity strength derived from anatomical variation, the gray matter (GM) volume was considered an important covariate in the functional synchrony analysis (Xie et al., 2015). An optimized voxel-based morphometry (VBM) analysis was conducted using SPM8 to calculate the GM volume in all subjects. The T1-weighted images were segmented to GM, WM, and CSF, and the segmented GM was subsequently normalized and smoothed with a 6-mm FWHM Gaussian kernel. The GM volume was regressed out as a covariate to control the effects on functional synchrony strength.

### Voxel-wise based functional connectivity analysis

The bilateral NAc were selected as regions of interest (ROIs) from the Harvard-Oxford subcortical atlas (Desikan et al., 2006) using REST software version 1.8 (http://www.restfmri.net). For each seed region, a voxel-wise functional connectivity analysis was performed using REST. The average time course in each NAc region, as the seed time course, was correlated with the time courses for all brain voxels using Pearson's cross correlation to obtain the correlation coefficient (CC). Fisher's Z-transformation was subsequently applied to improve the normality of the correlation coefficient (m = 0.5 ln[1 + CC]/[1 − CC]) (Lowe et al., 1998). Thus, each individual NAFC network map was obtained.

### Statistical analysis

#### Behavior analysis

Two independent sample *t*-tests and chi-square tests (only for gender) were performed for the demographic and behavior data comparison between the two groups (SPSS 20.0; SPSS Inc., Chicago, IL, USA). Partial correlation analysis was employed to detect the relationship between MGPS (also individual DA variants) and depression traits (including HAMD total score and sub-factor scores) in the MDD group after removing the effects of covariates including gender, age, education, and GM volumes. The significance level was set at *P* < 0.05, and the multiple comparison correction was conducted using Bonferroni adjustment. We also used the Kolmogorov–Smirnov Test to confirm that our data were normally distributed (*P* > 0.05).

#### R-fMRI connectivity analysis

First, a one-sample *t*-test was used to obtain an individual NAFC network pattern in each group (Figure S1). Cluster-level thresholds corrected for multiple comparisons were derived using a Monte Carlo simulation of random noise distribution in the present data, after correction using the 3dClustSim program in AFNI_16.3.00 (Feb. 2016) (GM mask correction (67,541 voxels), voxel level *P* < 0.05, cluster level α < 0.001, κ > 207, cluster size > 5535 mm^3^; https://afni.nimh.nih.gov/pub/dist/doc/program_help/3dClustSim.html). Second, multivariate linear regression analysis was employed to investigate the potential effects of disease (D), MGPS, and D × MGPS on the NAFC networks (3dRegAna, AFNI), after controlling for covariates of no interest including hemisphere, gender, age, education, and GM volumes. The statistical threshold was set at *P* < 0.01 (3dClustSim corrected, cluster level α < 0.001, κ > 54, cluster size > 1458 mm^3^). The following equation is the multivariate regression analysis for identifying the neural correlates of disease, MGPS, and interaction of disease and MGPS (D × MGPS) among all subjects:

$$\begin{array}{l} m_{i}=\;\beta_{0}+\beta_{1}\;\times \;D+\beta_{2}\times \text{MGPS}+\beta_{3}\;\times \;\left(D\times MGPS\right)+\beta_{4}\\ \times \;Gender+\beta_{5}\;\times \;Edu+\beta_{6}\;\times \;GM+\beta_{7}\;\times \;Age+\epsilon \end{array}$$

where m_*i*_ is the Z value of the *i*th voxel across all subjects; β_0_ is the intercept of the straight line fitting in the model; β_1_, β_2_, and β_3_ are the main effects of disease, MGPS and the D × MGPS interaction on the functional connectivity strength of the *i*th voxel on the NAFC networks; and β_4_, β_5_, β_6_, and β_7_ are the main effects of gender, education, GM volume, and age, respectively, which were discarded as covariates of no interest in the above linear regression model. The error term ϵε is assumed to have a Gaussian distribution and to be uncorrelated across subjects.

Third, to calculate the relative variance of the reward network explained by the MGPS, the average functional connectivity strength in each ROI from the main effect of MGPS on NAFC was extracted and then entered into linear and stepwise regression models after controlling for the effects of gender and group.

#### Mediation analysis

Mediation analysis was performed to explore the potential relationships among the MGPS, intrinsic NAFC, and behavior (depressive symptoms). Because of the observed significant correlation between MGPS and HAMD anxiety/somatization (HAMD-a) score in the MDD group, we investigated whether NAFC strength could mediate the effect of MGPS on HAMD-a in MDD patients. We used a simple moderation model from PROCESS Marco in SPSS for the mediation analysis (model 4) (Hayes, 2013). This model is described detailed in our previous study (Gong et al., 2017b). Briefly, it is based on 10,000 bootstrap samples for a bias-corrected bootstrap confidential interval (CI). The indirect effect is considered significant when the 95% CI does not include zero (with a null hypothesis that there is no indirect effect). Three-step regression models were constructed, as shown below:

(1) $$\begin{array}{l} \text{Y}=\text{cX}+{\text{c}}_{1}{\text{U}}_{1}{+\text{c}}_{2}{\text{U}}_{2}+{\text{c}}_{2}{\text{U}}_{2}+\text{e}1 \end{array}$$

(2) $$\begin{array}{l} \text{M}=\text{aX}+{\text{a}}_{1}{\text{U}}_{1}{+\text{a}}_{2}{\text{U}}_{2}{+\text{a}}_{2}{\text{U}}_{2}+\text{e}2 \end{array}$$

(3) $$\begin{array}{l} \text{Y}=c'\text{X}+\text{bM}+{\text{b}}_{1}{\text{U}}_{1}{+\text{B}}_{2}{\text{U}}_{2}{+\text{b}}_{2}{\text{U}}_{2}+\text{e}2 \end{array}$$

where X is the independent variable (MGPS), Y is the dependent variable (HAMD-a), M is the mediator (NAFC in each ROI of interactive effect of disease and MGPS), with each entered separately. U_1_, U_2_, and U_3_ are gender, education, and age, respectively. The *direct effect* is the effect of X on Y independent of the effect of M on Y (path *c'*). The *direct effect* between X and Y is not a necessary prerequisite for mediation (Hayes, 2009). The *indirect effect*, or the effect of X on Y via M, is estimated as the product of the effect of X on M and the effect of M on Y, controlling for X (*ab* with 95% bootstrap CI). The total effect of X on Y is the sum of the direct and indirect effects (path *c*).

## Results

### Demographic information and neuropsychiatric data

No significant differences were observed in the age, gender, education, or MGPS between the two groups. The MDD patients had smaller GM volumes (*P* = 0.041) and higher HAMD and HAMA scores compared with the CN subjects (*P* < 0.001) (Table 1). There were no significant differences in genotype or allele frequency in the four gene loci between the two groups (Table S1).

**Table 1:** Demographic and neuropsychiatric characteristics.

	**CN (*n* = 37)**	**MDD (*n* = 53)**	***T/X*^2^**	***P*-value**
Age	42.19 ± 11.49	39.81 ± 11.64	0.96	0.340
Gender (M/F)	21/16	26/27	0.52	0.524^†^
Education	11.54 ± 4.07	9.98 ± 4.00	1.81	0.074
GM (ml)	649.34 ± 64.16	622.59 ± 57.06	2.08	0.041
HAMD	1.11 ± 1.51	22.69 ± 4.69	26.83	<0.001
HAMA	1.41 ± 2.08	18.77 ± 6.05	16.75	<0.001
MGPS	1.57 ± 0.68	1.55 ± 0.69	0.07	0.941

† *, P-value was obtained by a chi-square test; CN, cognitively normal; MDD, major depressive disorder; GM, gray matter; HAMD, Hamilton Depression Rating Scale (17-item); HAMA, Hamilton Anxiety Rating Scale; MGPS, multilocus genetic profiles scores*.

### Relationship between MGPS and depression traits in MDD patients

Partial correlation analysis revealed that the MGPS was negatively correlated with HAMD-a score (*r* = −0.376, *P* = 0.013), but this association was not significant after Bonferroni correction. We did not find any other significant correlations between individual DA variants and the HAMD score, or between MGPS and the HAMD total and sub-factor scores (all *P* > 0.05, Table S2).

### Main effects of disease on the NAFC network

**Figure 1:**
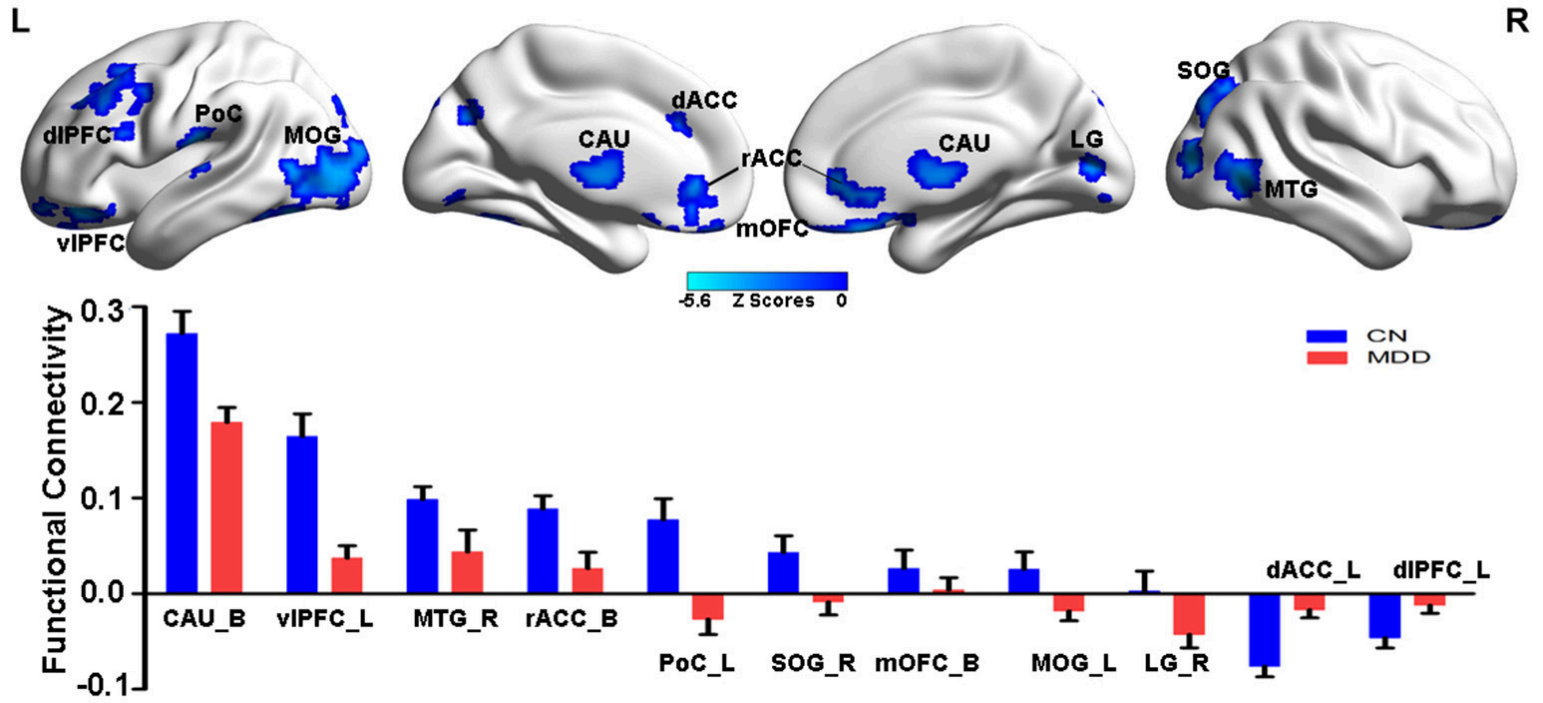
Group-level differences in NAFC network between CN and MDD subjects. **Top**: Brain regions with decreased NAFC in MDD patients compared with the CN group. Blue color indicates decreased connectivity in MDD patients. **Bottom**: Numerical representation of significant differences in NAFC strength between the two groups. CN, cognitively normal; MDD, major depressive disorder; NAFC, nucleus accumbens functional connectivity; CAU_B, bilateral caudate; vlPFC_L, left ventrolateral prefrontal cortex; MTG_R, right middle temporal gyrus; rACC_B, bilateral rostral anterior cingulate cortex; PoC_L, left postcentral cortex; SOG_R, right superior occipital gyrus; mOFC_B, bilateral medial orbital frontal cortex; MOG_L, left middle occipital gyrus; LG_R, right lingual gyrus; dACC_L, left dorsal anterior cingulate cortex; dlPFC_L, left dorsolateral prefrontal cortex.

Compared with CN subjects, MDD patients showed decreased positive and negative connectivity within the NAFC network. Specially, brain regions with decreased positive NAFC were primarily located in the bilateral caudate, rostral anterior cingulate cortex (rACC), medial orbital frontal cortex, left ventrolateral prefrontal cortex (vlPFC), postcentral cortex (PoC), middle occipital gyrus (MOG), right middle temporal cortex (MTG), and superior occipital gyrus (SOG). Brain regions with decreased negative connectivity were mainly in the left dorsal anterior cingulate cortex (dACC) and dorsolateral prefrontal cortex (dlPFC) (Figure 1 and Table S3).

### Main effects of MGPS on the NAFC network

**Figure 2:**
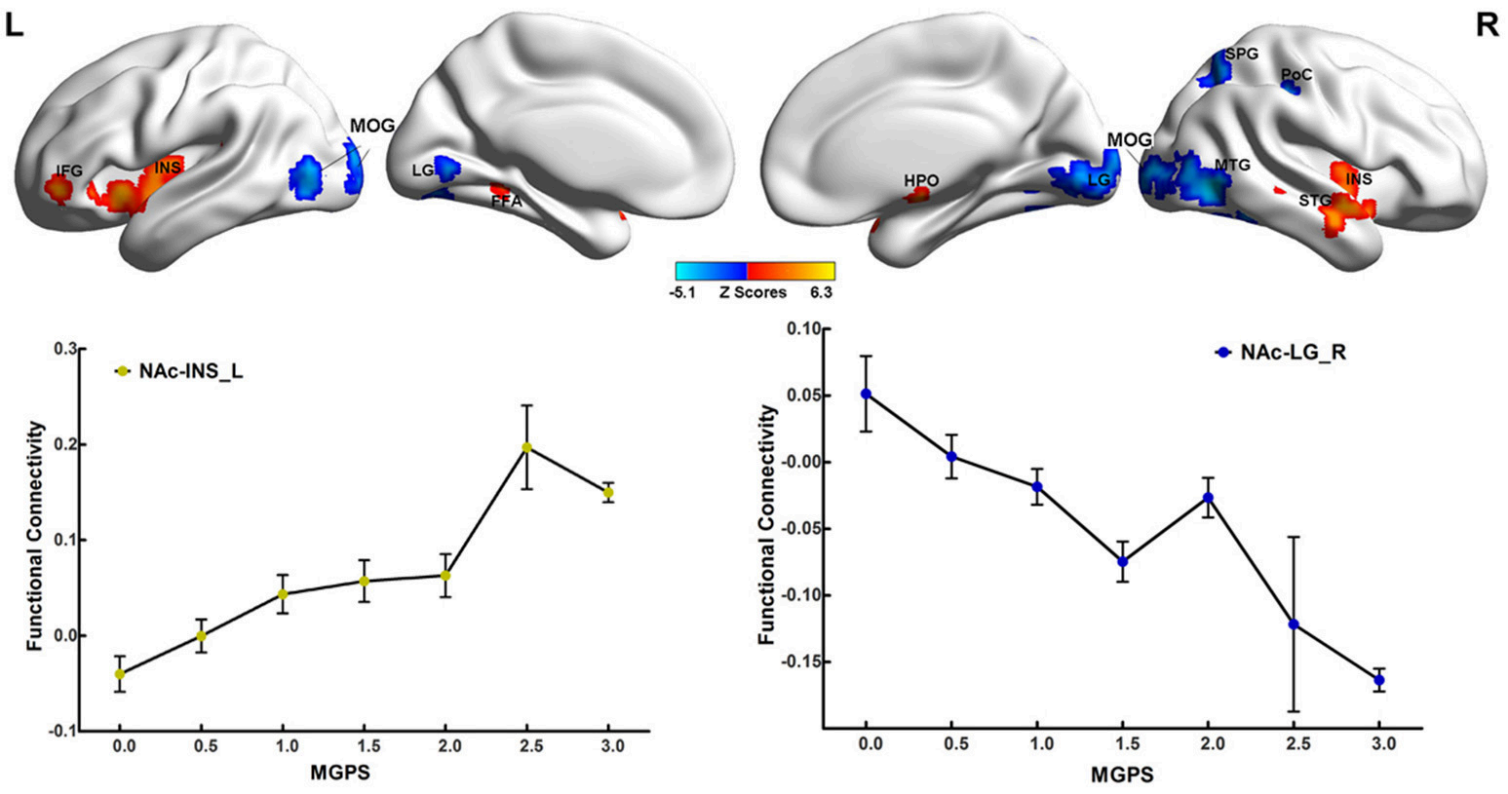
Main effects of MGPS on the NAFC network across all subjects. **Top**: The result illustrates significantly correlated regions between the MGPS and NAFC network among all subjects. Bright color indicates a positive correlation, while blue color indicates a negative correlation. **Bottom**: The representation of positive and negative correlations between MGPS and NAFC strength among all subjects was plotted. MGPS, multilocus genetic profile scores; NAFC, nucleus accumbens functional connectivity; INS, insular lobe; IFG, inferior frontal gyrus; FFA, fusiform area; HPO, hypothalamus; MTG, middle temporal gyrus; MOG, middle occipital gyrus; LG, lingual gyrus; SPG, superior parietal gyrus; PoC, postcentral cortex.

As illustrated in Figure 2 and Table S4, the MGPS was associated with both positive and negative effects on the NAFC network. Specifically, the positively correlated regions were located in the bilateral insular lobe, left inferior frontal gyrus (IFG), fusiform area (FFA), right hypothalamus, and superior temporal gyrus (STG), while the negatively correlated regions included the bilateral MOG and LG, right middle temporal gyrus (MTG), superior parietal gyrus (SPG), and PoC. Moreover, the linear regression models revealed that the MGPS accounted for 7.5–19.7% of all variability in these ROIs in the NAFC network. These contributions were still significant after multiple comparison correction (Bonferroni correction, significance was set at *P* < 0.05/10 = 0.005, see Table S4).

### Interactive effects between disease and MGPS on the NAFC network

**Figure 3:**
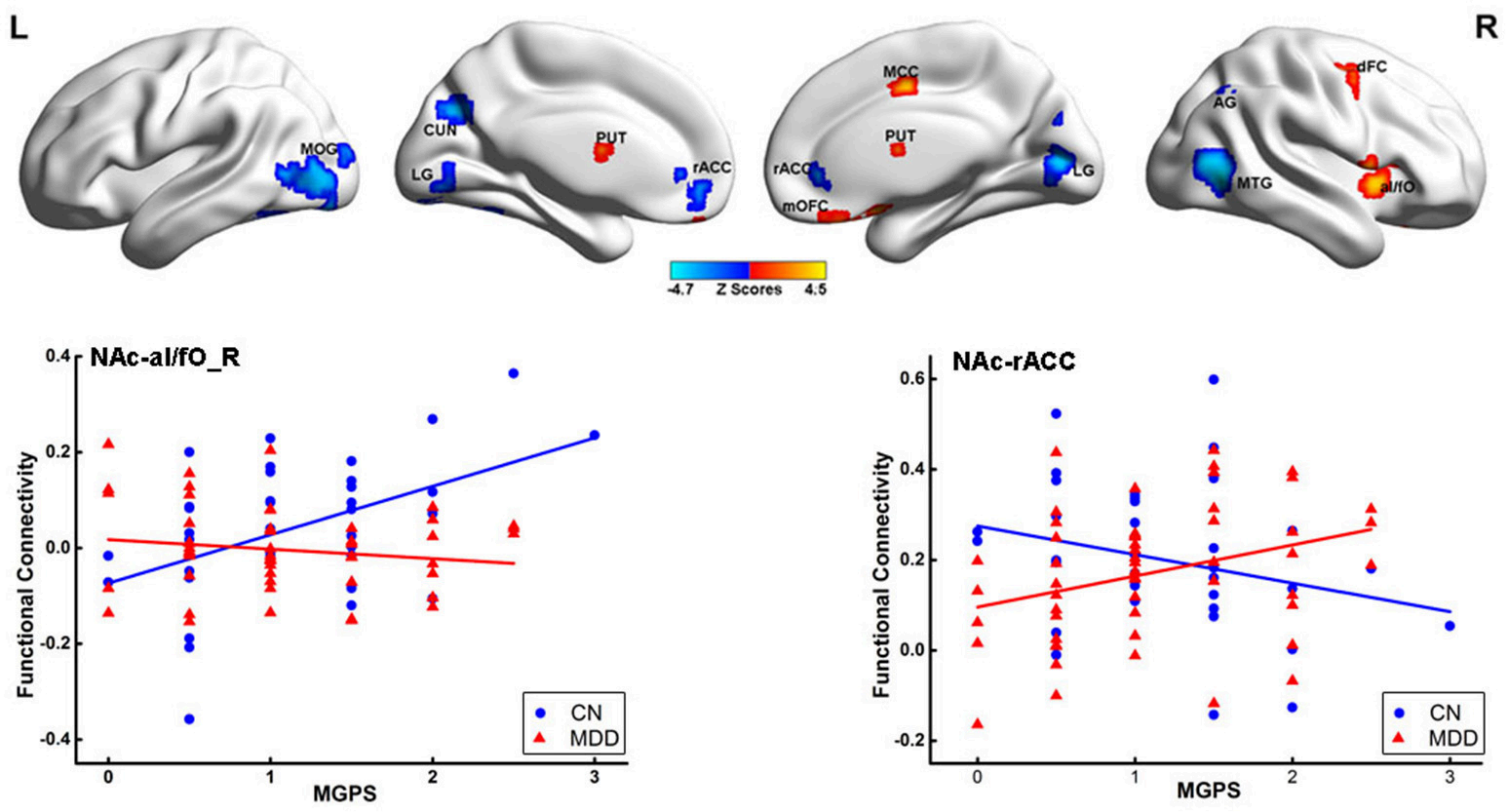
Interaction effects between disease and MGPS on the NAFC network. **Top:** Brain regions with significant interaction effects between disease and MGPS on the NAFC network. The bright color indicates that the MGPS and disease synergistically influenced the NAFC network, and blue color indicates that the MGPS and disease oppositely influenced the NAFC network. **Bottom:** Two-dimensional scatterplots illustrating the different correlation patterns on the representative regions with interaction effects of MGPS and disease on the NAFC network in CN and MDD patients, respectively. MGPS, multilocus genetic profile scores; NAFC, nucleus accumbens functional connectivity; PUT, putamen; al/fO, anterior insular/frontal opercula; MCC, middle cingulate cortex; dFC, dorsal frontal cortex; mOFC, medial orbital frontal cortex; LG, Lingual gyrus; MTG, middle temporal gyrus; rACC, rostral anterior cingulate cortex; MOG, middle occipital gyrus; CUN, cuneus; AG, angular gyrus.

Significant interactions between disease and MGPS were demonstrated in the NAFC network including the bilateral putamen, rACC, LG, left cuneus, MOG, right MTG, middle cingulate cortex, mOFC, angular gyrus (AG), anterior insular/frontal opercula (aI/fO), and dorsal frontal cortex (dFC) (Table S5). As displayed in Figure 3, MGPS and disease synergistically influenced the NAFC network across all subjects. Intriguingly, MGPS effects on the reward network showed opposite correlation patterns in MDD patients and CN subjects.

### Mediation analysis results

**Figure 4:**
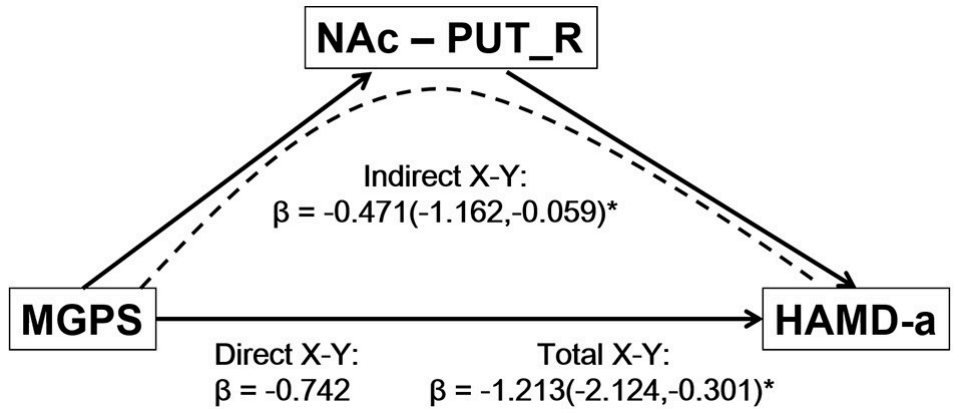
Result of mediation analysis. Mediation analysis revealed that the NAc-PUT_R connectivity negatively mediates the relationship between the MGPS and HAMD-a in MDD patients. MGPS, multilocus genetic profile score; HAMD-a, 17-item Hamilton Rating Scale for Depression anxiety/somatization factor; NAc, nucleus accumbens; PUT_R, right putamen; MDD, major depressive disorder.

The mediation analysis revealed a significant indirect effect of the NAc-putamen FC on the relationship between MGPS and HAMD-a performance in MDD patients. As shown in Figure 4, the functional connectivity of the NAc and right putamen negatively mediated the effects of the MGPS on HAMD-a in the MDD patients (indirect effect, β = −0.471, 95% CI = [−1.162, −0.059]). Namely, higher MGPS in patients with a higher NAFC in putamen might predict lower HAMD-a scores. No mediating effects of other ROIs were found in the mediation analysis after Bonferroni adjustment.

## Discussion

Our findings demonstrate the cumulative effects of multiple gene polymorphisms in the dopaminergic pathway on both behavior and the intrinsic reward network in MDD patients. First, the DA-MGPS is associated with anxiety/somatization symptoms in MDD patients. Second, DA-MGPS widely influences the NAFC network and accounts for 7.5–19.7% of all variability of the NAFC network. Third, depression and DA-MGPS synergistically contribute to the dysfunctional reward network, especially in fronto-striatal circuits. Fourth, the relationship between the MGPS and HAMD anxiety/somatization factor is mediated by NAFC in MDD patients, suggesting that reward-related intrinsic connectivity could modulate the DA-MGPS effect on depression traits. These findings provide novel insight into the polygenic cumulative role of the dopaminergic pathway in the reward network in depression patients.

First, the DA-MGPS was significantly negatively correlated with the anxiety/somatization factor in the MDD group, suggesting that a lower score might predict higher levels of anxious depression. Individuals with a DA-MAGPS above seven in the HAMD anxiety/somatization factor would be considered as anxious depression patients (Fava et al., 2008). Moreover, 46–81% of MDD patients present anxious depression, subsequently leading to melancholic features, more severe symptoms, greater risk of suicide, and lower antidepressant response (Maurizio Fava et al., 2006, 2008; Lin et al., 2014). Although a previous study found an association between DA genetic risk scores and the HAMD total score in 1,267 depressed subjects (Pearson-Fuhrhop et al., 2014), it is not clear whether multiple genes in the dopaminergic pathway contribute to the underlying neuropathology of anxious depression. The present study provides indirect evidence that the DA genetic profile is involved in anxious depression.

Second, consistent with previous task-related fMRI studies, NAFC was decreased in MDD patients, and disruption was primarily located in the fronto-striatal pathway (Tremblay et al., 2005; Pizzagalli et al., 2009). These brain regions are closely linked to the constructs of reward valuation and anticipation, as well as motivation (Der-Avakian and Markou, 2012; Berridge and Kringelbach, 2015). In addition, the caudate, mOFC, and adjacent rACC are important hubs for reward circuits that contribute to processing reward valuation, subjective pleasure, and reward anticipation (Kringelbach et al., 2003; Georgiadis and Kringelbach, 2012). Moreover, the dlPFC, vlPFC, and dACC are involved in emotion regulation, evaluation of the self, and executive function (Northoff and Bermpohl, 2004; Smith et al., 2009). Therefore, for the important role of the fronto-striatal pathway in reward and cognitive control process, decreased NAFC in this pathway indicates that MDD patients might suffer from an attenuated reward experience and decreased cognitive control during the resting state.

Third, it is not surprising that the DA-MGPS was associated with a widespread effect on the intrinsic NAFC network including the frontal, temporal, parietal, occipital, and insular lobes. Specially, the DA-MGPS is positively correlated with functional connectivity between the NAc and hypothalamus. Both the hypothalamus and NAc play core roles in reward and motivation, especially for food “liking” and “wanting” (Aston-Jones et al., 2010), and their interaction may contribute to maintaining homeostasis and mediating affect and motivation (Castro et al., 2015). Though the sample is relatively small, we also found significant variability (7.5–19.6%) in the reward network between individuals that was explained by the DA-MGPS. These findings support the important and extensive role of DA in the reward network.

Fourth, although there was no difference in DA-MGPS between two groups, opposite patterns of MGPS effect on reward network were observed between groups. The different patterns between the CN and MDD groups might indicate that the DA-MGPS effect on reward network would be non-linear. Several studies reported an inverted U-shaped relationship between DA signaling and fronto-striatal brain activity and cognitive performance in healthy participants and subjects with Parkinson's disease (Bertolino et al., 2009; Cools and D'Esposito, 2011; Fallon et al., 2013). Our recent published work also revealed a non-linear modulation of the interaction between the *COMT* gene and depression on brain function (Gong et al., 2017a). Interestingly, the interaction regions identified in the present study included the mOFC, rACC, and putamen, which functionally encompass the fronto-striatal pathway, and these are implicated in reward anticipation, valuation, motivation, and attainment (Dillon et al., 2014). Our findings verify and expand the notion of a non-linear effect of DA signaling on the fronto-striatal pathway at a DA polygene level. According to the consistency of dysfunctional NAFC and the non-linear effect of DA-MGPS on the fronto-striatal pathway, we hypothesize that dysfunction in the reward network and a lower DA genetic profile make subjects vulnerable to depression.

Importantly, mediation analysis showed that the link between the DA-MGPS and HAMD-a score was mediated through functional connectivity between the NAc and putamen. This extends our observation of a link among the DA genetic profile, reward circuit activity, and depression traits in healthy adults and MDD patients (Felten et al., 2011; Nikolova et al., 2011; Stice et al., 2012; Pearson-Fuhrhop et al., 2014), and implies that NAc-putamen connectivity may play a substantial role in DA gene effects on anxious depression in MDD patients. The NAc and putamen are substructures of the striatum and play pivotal roles in regulating dopamine, thus mediating the responses induced by both stress and reward (Cabib and Puglisi-Allegra, 2012; Foti et al., 2014; Stringaris et al., 2015). A recent study reported that remitted MDD patients exhibited potentiated striatal reactivity during a psychological stress task (Admon et al., 2015). Another study found that the robust reward responsiveness in the NAc may protect against the depressogenic effects of stress (Nikolova et al., 2012). These previous findings leave open the possibility that the observed relationship between the DA genetic profile and anxious depression is driven by weaker striatal functional coupling due to chronic psychological stress in MDD patients. From this view, our findings suggest that local striatal functional connectivity could be a predictor of anxious depression in individuals with lower DA-MGPS.

The present study also has several limitations. First, because participant genetic information came from a large research sample that assayed only 14,398 SNPs in the exome region, several DA genetic variants of interest were not genotyped, such as *DAT1* VNTR and *DRD2* Taq1A (Brody et al., 2006). Additional DA pathway genes should be covered to generalize our findings. Second, we did not perform neuropsychological tests to measure reward or motivation. Future studies should include a detailed reward estimate test combining specific tasks to further clarify the relationship between DA genetic profile and reward processing in MDD patients. Third, the small sample size and mixed sample (both inpatients and outpatients, first onset and recurrent) of this study limit the generalizability of the current result. Lastly, the method of MGPS was often used and averaged each SNP equally and additively to influence DA signaling and brain function. A more effective approach is necessary to provide a polygenic risk score (Bogdan et al., 2017). However, we still believe that the current study provides a useful starting point for multilocus genetic imaging investigations for MDD.

In summary, our findings show that the DA-MGPS specifically contributes to the reward network in patients with MDD. In addition, NAc-putamen functional connectivity mediates the relationship between the DA genetic profile and anxious depression. These findings provide new insight to guide further exploration into the polygenic effects on depression risk variants and may help reveal the complex gene–brain–behavior relationship.

## Author contributions

LG and CX: Conceived the study, designed and conducted the experiments, analyzed the data, and wrote the manuscript; CH, QY, YYY, and HW: Conducted the experiments, analyzed the data, and wrote the manuscript; HSZ, HXZ, and LL: Analyzed the data and wrote the manuscript; ZZ, YGY, and FB: Conceived the study, designed the experiments, analyzed the data, supervised the project, and wrote the manuscript.

### Conflict of interest statement

The authors declare that the research was conducted in the absence of any commercial or financial relationships that could be construed as a potential conflict of interest.

## Supplementary material

Table S1 Composition and distribution of multilocus genetic profile scores. DA, dopamine; CN, cognitively normal; MDD, major depressive disorder; *DRD2*, dopamine receptor D2; *DRD3*, dopamine receptor D3; *COMT*, Catechol-O-methyltransferase; *MAOA*, monoamine oxidase A.

Table S2 Adjusted association between MGPS/individual dopamine variants and depression traits in MDD group. MGPS, multilocus genetic profile score; HAMD, Hamilton Rating Scale for Depression; HAMD-a, HAMD anxiety/somatization factor; HAMD-w, HAMD weight factor; HAMD-c, HAMD cognitive disturbance factor; HAMD-r, HAMD retardation factor; HAMD-s, HAMD sleep disruption factor.

Table S3 Main effects of disease on NAFC networks. NAFC, nucleus accumbens functional connectivity; BA, Brodmann's area; MNI, Montreal Neurological Institute space; L, left; R, right; B, bilateral; NFC, nucleus accumbens functional connectivity; CAU, caudate; vlPFC, ventrolateral prefrontal cortex; MTG, middle temporal gyrus; rACC, rostral anterior cingulate cortex; PoC, postcentral cortex; SOG, superior occipital gyrus; mOFC, medial orbital frontal cortex; MOG, middle occipital gyrus; LG, lingual gyrus; dACC, dorsal anterior cingulate cortex; dlPFC, dorsolateral prefrontal cortex.

Table S4 Main effects of MGPS on NAFC networks in all subjects. MGPS, multilocus genetic profiles scores. NAFC, nucleus accumbens functional connectivity; BA, Brodmann's area; MNI, Montreal Neurological Institute space; L, left; R, right; B, bilateral; INS, insular lobe; IFG, inferior frontal gyrus; FFA, fusiform area; HPO, hypothalamus; MTG, middle temporal gyrus; MOG, middle occipital gyrus; LG, lingual gyrus; SPG, superior parietal gyrus; PoC, postcentral cortex.

Table S5 Interactive effects of group and MGPS on NAFC networks. MGPS, multilocus genetic profile scores; NAFC, nucleus accumbens functional connectivity; BA, Brodmann's area; MNI, Montreal Neurological Institute space; L, left; R, right; B, bilateral; PUT, putamen; al/fO, anterior insular/frontal opercula; MCC, middle cingulate cortex; mOFC, medial orbital cortex; dFC, dorsal frontal cortex; LG, lingual gyrus; MTG, middle temporal gyrus; rACC, rostral anterior cingulate cortex; MOG, middle occipital gyrus; CUN, cuneus; AG, angular gyrus.

Figure S1 Resting-state functional connectivity patterns of bilateral NAFC networks across all subjects (*P* < 0.05, 3dClustSim corrected). The results illustrate the different neural constructs of bilateral NAFC networks for CN and MDD subjects by using one sample *T*-test. Bright color indicates positive connectivity and blue color indicates negative connectivity. Color bar is presented with Z scores. NAFC, nucleus accumbens functional connectivity; CN, cognitively normal; MDD, major depressive disorder.
