# Supplementary material for: Mediating Role of the Reward Network in the Relationship between the Dopamine Multilocus Genetic Profile and Depression
Source: Front Mol Neurosci. 2017 Sep 14;10:292. doi: 10.3389/fnmol.2017.00292 (PMC5603675; doi:10.3389/fnmol.2017.00292)
Supplement: Table S1 — Composition and distribution of multilocus genetic profile scores. DA, dopamine; CN, cognitively normal; MDD, major depressive disorder; DRD2, dopamine receptor D2; DRD3, dopamine receptor D3; COMT, Catechol-O-methyltransferase; MAOA, monoamine oxidase A. [file Table1.DOCX]

**Table S1. Composition and distribution of multilocus genetic profile scores.**

| **Polymorphism** | **Genotypes** | **DA profile score** | **CN** | **MDD** |
| --- | --- | --- | --- | --- |
| ***DRD2* C957T**  **(rs6277)** | C/C | 0 | 31 | 46 |
|  | C/T | 0.5 | 6 | 7 |
| ***DRD3* Ser9Gly**  **(rs6280)** | Ser/Ser | 0 | 20 | 29 |
|  | Ser/Gly | 0.5 | 12 | 21 |
|  | Gly/Gly | 1 | 5 | 3 |
| ***COMT* Val^158^Met**  **(rs4680)** | Val/Val | 0 | 18 | 27 |
|  | Val/Met | 0.5 | 17 | 22 |
|  | Met/Met | 1 | 2 | 4 |
| ***MAOA* Arg^297^Arg**  **(rs6323)** | G/G | 0 | 13 | 20 |
|  | G/T | 0.5 | 11 | 16 |
|  | T/T | 1 | 13 | 17 |

Abbreviations: DA, dopamine; CN, cognitively normal; MDD, major depressive disorder; *DRD2*, dopamine receptor D2; *DRD3*, dopamine receptor D3; *COMT*, Catechol-O-methyltransferase; *MAOA*, monoamine oxidase A.
