# Supplementary material for: Mediating Role of the Reward Network in the Relationship between the Dopamine Multilocus Genetic Profile and Depression
Source: Front Mol Neurosci. 2017 Sep 14;10:292. doi: 10.3389/fnmol.2017.00292 (PMC5603675; doi:10.3389/fnmol.2017.00292)
Supplement: Table S2 — Adjusted association between MGPS/individual dopamine variants and depression traits in MDD group. MGPS, multilocus genetic profile score; HAMD, Hamilton Rating Scale for Depression; HAMD-a, HAMD anxiety/somatization factor; HAMD-w, HAMD weight factor; HAMD-c, HAMD cognitive disturbance factor; HAMD-r, HAMD retardation factor; HAMD-s, HAMD sleep disruption factor. [file Table2.DOCX]

**Table S2. Adjusted association between MGPS/individual dopamine variants and depression traits in MDD group**

|  |  | **HAMD** | **HAMD-a** | **HAMD-w** | **HAMD-c** | **HAMD-r** | **HAMD-s** |
| --- | --- | --- | --- | --- | --- | --- | --- |
| **MGPS** | *r* | -0.161 | -0.376 | 0.140 | -0.009 | 0.079 | 0.054 |
|  | *P* | 0.304 | **0.013** | 0.369 | 0.954 | 0.614 | 0.733 |
| **rs6277** | *r* | 0.057 | 0.051 | 0.057 | -0.025 | 0.166 | -0.036 |
|  | *P* | 0.702 | 0.732 | 0.702 | 0.866 | 0.266 | 0.810 |
| **rs6280** | *r* | -0.164 | -0.175 | -0.245 | 0.056 | -0.066 | -0.202 |
|  | *P* | 0.270 | 0.240 | 0.096 | 0.707 | 0.660 | 0.174 |
| **rs4680** | *r* | -0.095 | 0.015 | 0.169 | -0.187 | -0.102 | -0.189 |
|  | *P* | 0.524 | 0.921 | 0.255 | 0.208 | 0.496 | 0.204 |
| **rs6323** | *r* | 0.023 | -0.048 | 0.019 | 0.141 | 0.138 | 0.103 |
|  | *P* | 0.877 | 0.748 | 0.901 | 0.343 | 0.353 | 0.490 |

Abbreviations: MGPS, multilocus genetic profile score; HAMD, Hamilton Rating Scale for Depression; HAMD-a, HAMD anxiety/somatization factor; HAMD-w, HAMD weight factor; HAMD-c, HAMD cognitive disturbance factor; HAMD-r, HAMD retardation factor; HAMD-s, HAMD sleep disruption factor.
