# Supplementary material for: Mediating Role of the Reward Network in the Relationship between the Dopamine Multilocus Genetic Profile and Depression
Source: Front Mol Neurosci. 2017 Sep 14;10:292. doi: 10.3389/fnmol.2017.00292 (PMC5603675; doi:10.3389/fnmol.2017.00292)
Supplement: Table S3 — Main effects of disease on NAFC networks. NAFC, nucleus accumbens functional connectivity; BA, Brodmann's area; MNI, Montreal Neurological Institute space; L, left; R, right; B, bilateral; NFC, nucleus accumbens functional connectivity; CAU, caudate; vlPFC, ventrolateral prefrontal cortex; MTG, middle temporal gyrus; rACC, rostral anterior cingulate cortex; PoC, postcentral cortex; SOG, superior occipital gyrus; mOFC, medial orbital frontal cortex; MOG, middle occipital gyrus; LG, lingual gyrus; dACC, dorsal anterior cingulate cortex; dlPFC, dorsolateral prefrontal cortex. [file Table3.DOCX]

**Table S3. Main effects of disease on NAFC networks**

| **Brain Region** | **Side** | **BA** | **Cluster size(mm^3^)** | **MNI Coordinate(RAI)** | | | **Peak Z scores** |
| --- | --- | --- | --- | --- | --- | --- | --- |
|  |  |  |  | X | Y | Z |  |
| **CAU** | B | - | 9990 | -6 | -3 | 6 | -3.64 |
| **vlPFC** | L | 11 | 2160 | -27 | 39 | -15 | -4.26 |
| **MTG** | R | 37 | 4401 | 57 | -72 | 9 | -4.51 |
| **rACC** | B | 32 | 2565 | 3 | 36 | -6 | -3.51 |
| **PoC** | L | 42 | 2484 | -63 | -15 | 9 | -4.39 |
| **SOG** | R | 19 | 2160 | 30 | -81 | 48 | -4.45 |
| **mOFC** | B | 11 | 7425 | 0 | 45 | -30 | -5.64 |
| **MOG** | L | 18 | 10287 | -54 | -75 | -3 | -4.92 |
| **LG** | R | 17 | 2646 | 33 | -90 | 9 | -4.04 |
| **dACC** | L | 32 | 2916 | -13 | 34 | 28 | -3.21 |
| **dlPFC** | L | 9 | 5022 | -33 | 9 | 36 | -3.75 |

Abbreviations: NAFC, nucleus accumbens functional connectivity; BA, Brodmann’s area; MNI, Montreal Neurological Institute space；L, left; R, right; B, bilateral; NFC, nucleus accumbens functional connectivity; CAU, caudate; vlPFC, ventrolateral prefrontal cortex; MTG, middle temporal gyrus; rACC, rostral anterior cingulate cortex; PoC, postcentral cortex; SOG, superior occipital gyrus; mOFC, medial orbital frontal cortex; MOG, middle occipital gyrus; LG, lingual gyrus; dACC, dorsal anterior cingulate cortex; dlPFC, dorsolateral prefrontal cortex.
