# Supplementary material for: Mediating Role of the Reward Network in the Relationship between the Dopamine Multilocus Genetic Profile and Depression
Source: Front Mol Neurosci. 2017 Sep 14;10:292. doi: 10.3389/fnmol.2017.00292 (PMC5603675; doi:10.3389/fnmol.2017.00292)
Supplement: Table S4 — Main effects of MGPS on NAFC networks in all subjects. MGPS, multilocus genetic profiles scores. NAFC, nucleus accumbens functional connectivity; BA, Brodmann's area; MNI, Montreal Neurological Institute space; L, left; R, right; B, bilateral; INS, insular lobe; IFG, inferior frontal gyrus; FFA, fusiform area; HPO, hypothalamus; MTG, middle temporal gyrus; MOG, middle occipital gyrus; LG, lingual gyrus; SPG, superior parietal gyrus; PoC, postcentral cortex. [file Table4.DOCX]

**Table S4. Main effects of MGPS on NAFC networks in all subjects**

| **Brain Region** | **Side** | **BA** | **Cluster size(mm^3^)** | **MNI Coordinate(RAI)** | | | **Peak Z scores** | ***R^2^*** | ***β*** | ***P*** |
| --- | --- | --- | --- | --- | --- | --- | --- | --- | --- | --- |
|  |  |  |  | X | Y | Z |  |  |  |  |
| **INS** | L | 47 | 6615 | -33 | 3 | -12 | 5.15 | 0.192 | 0.448 | <0.001 |
| **INS** | R | 48 | 810 | 42 | -3 | -3 | 3.56 | 0.105 | 0.339 | 0.001 |
| **IFG** | L | 47 | 999 | -45 | 45 | -6 | 3.69 | 0.089 | 0.315 | 0.002 |
| **FFA** | L | 37 | 1431 | -15 | -45 | -18 | 4.41 | 0.160 | 0.412 | <0.001 |
| **HPO** | R | - | 1188 | 9 | -15 | -24 | 3.87 | 0.090 | 0.317 | 0.002 |
| **MTG** | R | 21 | 1890 | 72 | -24 | -3 | 6.24 | 0.151 | 0.401 | <0.001 |
| **MOG/LG** | R | 18 | 15174 | 24 | -84 | 3 | -5.10 | 0.197 | -0.444 | <0.001 |
| **MOG/LG** | L | 19 | 3375 | -48 | -75 | 0 | -4.43 | 0.075 | -0.292 | 0.005 |
| **SPG** | R | 7 | 1296 | 30 | -66 | 51 | -3.67 | 0.077 | -0.296 | 0.005 |
| **PoC** | R | 2 | 1215 | 42 | -30 | 39 | -3.52 | 0.096 | -0.326 | 0.002 |

Abbreviations: MGPS, multilocus genetic profiles scores. NAFC, nucleus accumbens functional connectivity; BA, Brodmann’s area; MNI, Montreal Neurological Institute space；L, left; R, right; B, bilateral; INS, insular lobe; IFG, inferior frontal gyrus; FFA, fusiform area; HPO, hypothalamus; MTG, middle temporal gyrus; MOG, middle occipital gyrus; LG, lingual gyrus; SPG, superior parietal gyrus; PoC, postcentral cortex.
