# Supplementary material for: Mediating Role of the Reward Network in the Relationship between the Dopamine Multilocus Genetic Profile and Depression
Source: Front Mol Neurosci. 2017 Sep 14;10:292. doi: 10.3389/fnmol.2017.00292 (PMC5603675; doi:10.3389/fnmol.2017.00292)
Supplement: Table S5 — Interactive effects of group and MGPS on NAFC networks. MGPS, multilocus genetic profile scores; NAFC, nucleus accumbens functional connectivity; BA, Brodmann's area; MNI, Montreal Neurological Institute space; L, left; R, right; B, bilateral; PUT, putamen; al/fO, anterior insular/frontal opercula; MCC, middle cingulate cortex; mOFC, medial orbital cortex; dFC, dorsal frontal cortex; LG, lingual gyrus; MTG, middle temporal gyrus; rACC, rostral anterior cingulate cortex; MOG, middle occipital gyrus; CUN, cuneus; AG, angular gyrus. [file Table5.DOCX]

**Table S5. Interactive effects of group and MGPS on NAFC networks.**

| **Brain Region** | **Side** | **BA** | **Cluster size(mm^3^)** | **MNI Coordinate(RAI)** | | | **Peak Z scores** |
| --- | --- | --- | --- | --- | --- | --- | --- |
|  |  |  |  | X | Y | Z |  |
| **PUT** | L | - | 675 | -21 | -9 | 3 | 3.59 |
| **PUT** | R | - | 621 | 21 | 0 | 6 | 3.42 |
| **aI/fO** | R | 13 | 2484 | 39 | 18 | 12 | 3.91 |
| **MCC** | R | 6 | 783 | 12 | -3 | 42 | 3.81 |
| **mOFC** | R | - | 729 | 21 | 0 | 6 | 3.42 |
| **dFC** | R | 6 | 1593 | 30 | -3 | 48 | 4.05 |
| **LG** | R | 17 | 1404 | 12 | -78 | 3 | -3.73 |
| **MTG** | R | 37 | 7749 | 51 | -72 | 9 | -4.43 |
| **rACC** | B | 32 | 945 | -6 | 48 | -9 | -3.23 |
| **MOG** | L | 19 | 7101 | -51 | -75 | -3 | -4.69 |
| **CUN** | L | 7 | 1755 | -12 | -72 | 30 | -4.13 |
| **AG** | R | 7 | 1701 | 30 | -81 | 48 | -4.22 |

Abbreviations: MGPS, multilocus genetic profile scores; NAFC, nucleus accumbens functional connectivity; BA, Brodmann’s area; MNI, Montreal Neurological Institute space；L, left; R, right; B, bilateral; PUT, putamen; al/fO, anterior insular/frontal opercula; MCC, middle cingulate cortex; mOFC, medial orbital cortex; dFC, dorsal frontal cortex; LG, lingual gyrus; MTG, middle temporal gyrus; rACC, rostral anterior cingulate cortex; MOG, middle occipital gyrus; CUN, cuneus; AG, angular gyrus.
