# Supplementary figures and images for: Mediating Role of the Reward Network in the Relationship between the Dopamine Multilocus Genetic Profile and Depression
Source: Front Mol Neurosci. 2017 Sep 14;10:292. doi: 10.3389/fnmol.2017.00292 (PMC5603675; doi:10.3389/fnmol.2017.00292)

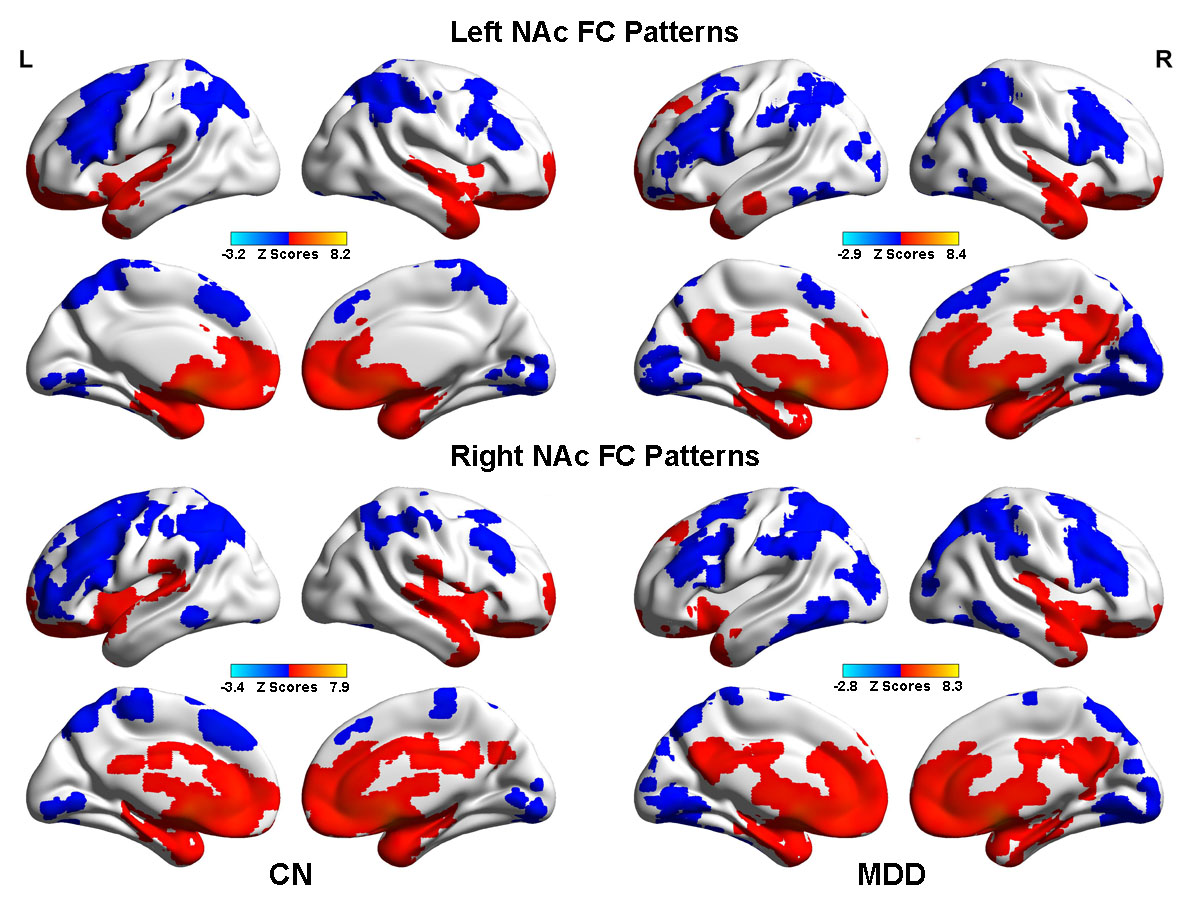

Supplement: Figure S1 — Resting-state functional connectivity patterns of bilateral NAFC networks across all subjects (P < 0.05, 3dClustSim corrected). The results illustrate the different neural constructs of bilateral NAFC networks for CN and MDD subjects by using one sample T-test. Bright color indicates positive connectivity and blue color indicates negative connectivity. Color bar is presented with Z scores. NAFC, nucleus accumbens functional connectivity; CN, cognitively normal; MDD, major depressive disorder. [file Image1.JPEG]
